# Supplementary material for: Vertical Transfer of Metabolites Detectable from Newborn’s Dried Blood Spot Samples Using UPLC-MS: A Chemometric Study
Source: Metabolites. 2022 Jan 20;12(2):94. doi: 10.3390/metabo12020094 (PMC8879569; doi:10.3390/metabo12020094)
Supplement: Supplementary file 1 [file metabolites-12-00094-s001.zip › metabolites-1546884-supplementary.pdf]

Supplementary tables and figures:

Table S1. Weakly transferred metabolites ($0.1 < R \leq 0.3$) with associated biochemical taxonomy and estimated correlations.

Biochemical	Taxonomy	$R_{m24\text{-}m1}$	$R_{m1\text{-}c}$	$R_{m24\text{-}c}$
beta-hydroxyisovaleroylcarnitine	Amino Acid	0.42	0.30	0.35
theobromine	Xenobiotics	0.26	0.27	0.26
arachidonoylcarnitine (C20:4)	Lipid	0.41	0.23	0.13
carnitine	Lipid	0.27	0.18	0.23
docosahexaenoate (DHA; 22:6n3)	Lipid	0.09	0.18	0.02
creatinine	Amino Acid	0.50	0.17	0.17
5.6-dihydrouridine	Nucleotide	0.36	0.17	0.09
hexanoylcarnitine (C6)	Lipid	0.29	0.16	0.18
1-methylhistidine	Amino Acid	0.03	0.15	0.10
N-acetylmethionine	Amino Acid	0.20	0.14	0.09
2-aminoheptanoate	Lipid	0.33	0.14	0.11
N2-acetyl.N6-methyllysine	Amino Acid	0.83	0.14	0.07
3-(4-hydroxyphenyl)lactate (HPLA)	Amino Acid	0.44	0.13	0.09
3-methoxytyrosine	Amino Acid	0.40	0.13	0.14
acetylcarnitine (C2)	Lipid	0.28	0.13	0.15
gamma-glutamylphenylalanine	Peptide	0.30	0.13	0.10
palmitoleoylcarnitine (C16:1)*	Lipid	0.16	0.13	0.07
phenylalanine	Amino Acid	0.25	0.13	0.10
arginine	Amino Acid	0.31	0.12	0.08
caproate (6:0)	Lipid	0.16	0.12	0.02
propionylcarnitine (C3)	Lipid	0.31	0.12	0.16
aconitate [cis or trans]	Energy	0.22	0.11	0.02
palmitoylcarnitine (C16)	Lipid	0.28	0.11	0.09
glutamine conjugate of C6H10O2 (1)*	Partially Characterized Molecules	0.30	0.11	0.07
citrate	Energy	0.27	0.11	0.04
cis-4-decenoylcarnitine (C10:1)	Lipid	0.25	0.11	0.02
N-acetylcarnosine	Amino Acid	0.54	0.11	0.05
metronidazole	Xenobiotics	0.00	0.11	-0.01

*: putative annotation.

Table S2. Non-transferred metabolites (R ≤ 0.1) with associated biochemical taxonomy and estimated correlations.

Biochemical	Taxonomy	$R_{m24\text{-}m1}$	$R_{m1\text{-}c}$	$R_{m24\text{-}c}$
5.6-dihydrothymine	Nucleotide	0.40	0.10	-0.04
hexanoylglutamine	Lipid	0.23	0.10	0.24
(R)-3-hydroxybutyrylcarnitine	Lipid	0.09	0.10	0.08
lysine	Amino Acid	0.30	0.09	0.04
theanine	Xenobiotics	0.54	0.09	0.04
2-aminooctanoate	Lipid	0.21	0.09	0.09
biliverdin	Cofactors and Vitamins	0.45	-0.09	-0.06
glutamine conjugate of C6H10O2 (2)*	Partially Characterized Molecules	0.23	0.09	0.00
linoleoylcarnitine (C18:2)*	Lipid	0.31	0.09	0.05
hydroxycotinine	Xenobiotics	0.75	-0.09	0.00
gamma-glutamylglutamate	Peptide	0.24	0.09	0.00
methyl glucopyranoside (alpha + beta)	Xenobiotics	0.63	-0.09	-0.02
3-methylcytidine	Nucleotide	0.61	0.09	0.06
gamma-glutamylglutamine	Peptide	0.26	-0.09	-0.01
suberoylcarnitine (C8-DC)	Lipid	0.22	0.09	0.02
linoleate (18:2n6)	Lipid	0.12	-0.08	-0.09
3-methoxycatechol sulfate (2)	Xenobiotics	0.29	0.08	0.03
lidocaine	Xenobiotics	0.03	0.08	0.01
octadecanedioylcarnitine (C18-DC)*	Lipid	0.44	0.08	0.02
thioproline	Xenobiotics	0.27	0.08	-0.01
undecenoylcarnitine (C11:1)	Lipid	0.25	0.08	0.14
tryptophan	Amino Acid	0.32	0.08	0.09
arabitol/xylitol	Carbohydrate	0.26	0.08	0.08
hydroxy-N6,N6,N6-trimethyllysine*	Amino Acid	0.24	0.08	0.09
N2,N2-dimethylguanosine	Nucleotide	0.56	0.07	0.13
3-hydroxyhexanoate	Lipid	0.10	0.07	-0.01
octadecenedioylcarnitine (C18:1-DC)*	Lipid	0.29	0.07	0.06
7-methylguanine	Nucleotide	0.59	0.07	0.16
gamma-glutamylvaline	Peptide	0.39	0.07	0.05
cysteine	Amino Acid	0.43	-0.07	-0.06
cortisol	Lipid	0.30	-0.07	-0.01
3,4-methyleneheptanoate	Xenobiotics	0.22	0.07	0.03
5-hydroxylysine	Amino Acid	0.13	-0.07	0.00
2-ketocaprylate	Amino Acid	0.10	0.07	0.03
myristoleoylcarnitine (C14:1)*	Lipid	0.07	0.07	0.04
isovalerylcarnitine (C5)	Amino Acid	0.32	0.07	-0.03
N-acetylphenylalanine	Amino Acid	0.62	0.07	0.12
nisinate (24:6n3)	Lipid	0.28	-0.07	-0.02
3-methylcrotonylglycine	Amino Acid	0.38	-0.06	-0.09
1-carboxyethylvaline	Amino Acid	0.22	0.06	0.05
dihydroorotate	Nucleotide	0.28	-0.06	-0.01
dimethylarginine (ADMA + SDMA)	Amino Acid	0.41	0.06	0.07
methionine sulfone	Amino Acid	0.65	0.06	0.02
N-acetyl-1-methylhistidine*	Amino Acid	0.61	0.06	0.03
3-hydroxybutyroylglycine	Lipid	0.21	-0.06	-0.06
tridecenedioate (C13:1-DC)*	Lipid	0.27	0.06	-0.03

succinylcarnitine (C4-DC)	Energy	0.48	0.06	0.17
prolylglycine	Peptide	0.19	0.06	0.06
gamma-glutamylglycine	Peptide	0.54	-0.06	-0.03
N-formylmethionine	Amino Acid	0.63	0.06	0.09
2-hydroxylaurate	Lipid	0.28	0.05	-0.01
4-guanidinobutanoate	Amino Acid	0.31	-0.05	0.00
alpha-ketoglutarate	Energy	0.44	0.05	0.04
spermine	Amino Acid	-0.01	0.05	0.02
2-hydroxypalmitate	Lipid	0.07	0.05	-0.01
glutamine	Amino Acid	0.36	0.05	0.05
2-hydroxyibuprofen	Xenobiotics	0.18	0.05	-0.07
4-hydroxyhippurate	Xenobiotics	0.17	-0.05	-0.02
3-methylhistidine	Amino Acid	0.02	0.05	0.02
6-hydroxyindole sulfate	Xenobiotics	0.41	-0.05	0.01
cytosine	Nucleotide	0.15	-0.05	-0.05
2-aminoadipate	Amino Acid	0.49	-0.05	-0.01
3-hydroxykynurenine	Amino Acid	0.37	0.05	0.03
glycine conjugate of C10H14O2 (1)*	Partially Characterized Molecules	0.33	0.05	0.03
7-HOCA	Lipid	0.27	0.05	0.07
3-(N-acetyl-L-cystein-S-yl) acetaminophen	Xenobiotics	-0.02	-0.05	0.01
N-acetyltryptophan	Amino Acid	0.86	-0.05	-0.03
2R,3R-dihydroxybutyrate	Lipid	0.33	0.05	0.02
propionylglycine (C3)	Lipid	0.48	0.05	-0.02
S-methylmethionine	Amino Acid	0.08	-0.05	-0.04
isovalerylglycine	Amino Acid	0.41	0.05	-0.02
iminodiacetate (IDA)	Xenobiotics	0.23	0.05	0.06
quinate	Xenobiotics	0.58	0.05	0.00
palmitoyl ethanolamide	Lipid	0.16	0.05	-0.04
guanosine	Nucleotide	-0.01	-0.05	0.00
(14 or 15)-methylpalmitate (a17:0 or i17:0)	Lipid	0.09	0.05	0.02
3-methyl-2-oxobutyrate	Amino Acid	0.30	0.05	-0.01
histidine	Amino Acid	0.24	-0.05	-0.05
proline	Amino Acid	0.30	0.05	0.02
gamma-glutamyl-2-aminobutyrate	Peptide	0.10	-0.04	0.02
5-methylthioadenosine (MTA)	Amino Acid	0.24	0.04	0.00
N6-acetyllysine	Amino Acid	0.48	0.04	0.01
N-acetylleucine	Amino Acid	0.18	-0.04	0.05
(2 or 3)-decenoate (10:1n7 or n8)	Lipid	0.12	0.04	0.03
4-hydroxy-2-oxoglutaric acid	Lipid	0.40	-0.04	0.01
3beta-hydroxy-5-cholestenoate	Lipid	0.33	-0.04	-0.02
alpha-ketobutyrate	Amino Acid	0.25	-0.04	-0.08
thymol sulfate	Xenobiotics	0.04	-0.04	0.00
phenyllactate (PLA)	Amino Acid	0.24	-0.04	-0.03
gamma-glutamylalanine	Peptide	0.22	-0.04	-0.06
hydroxyproline	Amino Acid	0.15	-0.04	0.02
N-acetyl-2-aminooctanoate*	Lipid	0.27	-0.04	0.00
metoprolol acid metabolite*	Xenobiotics	0.26	0.04	0.03
valylleucine	Peptide	0.04	0.04	0.01
allantoin	Nucleotide	0.27	-0.04	0.02

cinnamoylglycine	Xenobiotics	0.33	0.04	0.00
kynurenine	Amino Acid	0.32	0.04	0.02
alpha-hydroxyisocaproate	Amino Acid	0.23	-0.04	-0.08
N-formylanthranilic acid	Amino Acid	0.46	0.04	-0.03
leucine	Amino Acid	0.25	0.04	0.01
glycerol 3-phosphate	Lipid	0.19	-0.04	0.02
6-oxopiperidine-2-carboxylate	Amino Acid	0.36	0.04	-0.04
glycerophosphoethanolamine	Lipid	0.13	-0.04	0.04
choline	Lipid	0.26	0.04	0.05
oleoylcarnitine (C18)	Lipid	0.28	-0.04	0.03
spermidine	Amino Acid	0.00	0.04	0.04
dodecanedioate (C12)	Lipid	-0.01	-0.03	-0.02
citalopram propionate*	Xenobiotics	0.65	0.03	0.01
3-indoleglyoxylic acid	Xenobiotics	0.63	0.03	0.01
4-hydroxyphenylacetate	Amino Acid	0.39	0.03	-0.08
methyl indole-3-acetate	Xenobiotics	0.37	-0.03	0.03
deoxycholic acid (12 or 24)-sulfate*	Lipid	0.37	-0.03	0.11
2-hydroxy-4-(methylthio)butanoic acid	Amino Acid	0.25	-0.03	0.17
3-phenylpropionate (hydrocinnamate)	Xenobiotics	0.40	-0.03	-0.05
N6,N6,N6-trimethyllysine	Amino Acid	0.24	-0.03	0.03
gamma-glutamylhistidine	Peptide	0.28	-0.03	-0.02
2-hydroxyacetaminophen sulfate*	Xenobiotics	0.08	0.03	0.05
phenylalanylhydroxyproline*	Peptide	0.21	0.03	0.04
vanillactate	Amino Acid	0.10	0.03	-0.03
3-hydroxydecanoate	Lipid	0.09	-0.03	-0.01
glucuronate	Carbohydrate	0.30	-0.03	-0.01
glutarate (C5-DC)	Lipid	0.35	0.03	-0.03
indoleacetate	Amino Acid	0.43	0.03	0.02
pipecolate	Amino Acid	0.52	-0.03	-0.02
16-hydroxypalmitate	Lipid	0.13	-0.03	0.11
valine	Amino Acid	0.11	0.03	0.00
gamma-glutamyltyrosine	Peptide	0.19	-0.03	-0.07
N-oleoylserine	Lipid	0.42	0.03	0.04
dihydroferulate	Xenobiotics	0.23	-0.03	-0.02
butyrylcarnitine (C4)	Lipid	0.54	0.03	0.06
asparagine	Amino Acid	0.34	0.03	-0.01
threonine	Amino Acid	0.38	0.03	0.01
inosine 5'-monophosphate (IMP)	Nucleotide	0.15	0.03	0.08
3-amino-2-piperidone	Amino Acid	0.35	-0.03	0.05
3-methylglutaconate	Amino Acid	0.76	-0.03	-0.03
gamma-glutamylthreonine	Peptide	0.35	0.03	0.00
N1-Methyl-4-pyridone-3-carboxamide	Cofactors and Vitamins	0.23	-0.03	-0.01
alpha-ketoglutaramate*	Amino Acid	0.29	-0.03	0.02
betaine	Amino Acid	0.26	-0.03	-0.05
2-oxoarginine*	Amino Acid	0.26	0.03	0.03
pyridoxate	Cofactors and Vitamins	0.27	-0.03	0.06
6-bromotryptophan	Amino Acid	0.58	0.03	0.04
3-hydroxyhippurate	Xenobiotics	0.25	-0.03	0.03
methionine sulfoxide	Amino Acid	0.23	0.03	0.05

phosphoethanolamine (PE)	Lipid	0.24	0.03	-0.02
uracil	Nucleotide	0.13	-0.03	0.05
linolenate (18:3n3 or 3n6)	Lipid	0.50	-0.03	0.08
indolepropionate	Amino Acid	0.38	-0.03	0.19
N-palmitoylglycine	Lipid	0.20	-0.03	-0.01
2-piperidinone	Xenobiotics	0.33	0.02	0.08
N-palmitoylserine	Lipid	0.25	0.02	-0.02
glutamate	Amino Acid	0.26	0.02	0.04
1-methyladenosine	Nucleotide	0.49	0.02	0.04
homocitrulline	Amino Acid	0.18	0.02	0.01
3-formylindole	Xenobiotics	0.48	0.02	-0.03
glycine	Amino Acid	0.60	0.02	-0.02
citraconate/glutaconate	Energy	0.45	-0.02	0.02
N6,N6-dimethyllysine	Amino Acid	0.85	-0.02	-0.04
N,N-dimethylalanine	Amino Acid	0.53	-0.02	-0.05
tyrosine	Amino Acid	0.28	0.02	0.05
1,2,3-benzenetriol sulfate (2)	Xenobiotics	0.20	-0.02	-0.02
3-methylglutarylcarnitine (2)	Amino Acid	0.73	0.02	-0.01
picolinoylglycine	Lipid	0.14	-0.02	-0.01
1-myristoylglycerol (14:0)	Lipid	0.11	-0.02	-0.01
ornithine	Amino Acid	0.27	0.02	0.01
hydroxyasparagine	Amino Acid	0.63	-0.02	0.03
taurine	Amino Acid	0.24	0.02	0.06
N-acetyltheanine	Xenobiotics	0.13	0.02	0.09
indolelactate	Amino Acid	0.49	0.02	-0.04
N-acetylserine	Amino Acid	0.60	-0.02	-0.05
arachidonate (20:4n6)	Lipid	0.10	-0.02	0.07
1-carboxyethylphenylalanine	Amino Acid	0.19	-0.02	-0.09
heptenedioate (C7:1-DC)*	Lipid	0.29	0.02	-0.09
1,3,7-trimethylurate	Xenobiotics	0.44	-0.02	-0.01
N-acetylglucosamine/N-acetylgalactosamine	Carbohydrate	0.12	-0.02	-0.11
tartarate	Xenobiotics	0.21	-0.02	-0.06
caprate (10:0)	Lipid	0.09	-0.02	0.00
13-HODE + 9-HODE	Lipid	0.14	-0.02	0.08
3-hydroxyhexanoylcarnitine (1)	Lipid	0.14	0.02	0.05
N-acetylcitrulline	Amino Acid	0.58	-0.01	-0.03
21-hydroxypregnenolone disulfate	Lipid	0.67	0.01	-0.03
cortisone	Lipid	0.23	0.01	0.04
N6-carboxymethyllysine	Carbohydrate	0.27	-0.01	-0.04
erythritol	Xenobiotics	0.05	-0.01	-0.04
N,N,N-trimethyl-alanylproline betaine (TMAP)	Amino Acid	0.21	0.01	0.05
phenylpyruvate	Amino Acid	0.31	-0.01	-0.07
3-phosphoglycerate	Carbohydrate	0.09	0.01	0.10
12-HETE	Lipid	0.04	-0.01	-0.02
gluconate	Xenobiotics	0.46	0.01	-0.02
N-(2-furoyl)glycine	Xenobiotics	0.29	-0.01	0.10
creatine	Amino Acid	0.29	0.01	0.04
8-methoxykynurenate	Amino Acid	0.41	0.01	0.09
sphingosine	Lipid	0.31	-0.01	-0.04

palmitamide (16:0)	Lipid	0.45	-0.01	0.02
citrulline	Amino Acid	0.33	0.01	-0.02
indolin-2-one	Xenobiotics	0.47	-0.01	0.00
O-sulfo-L-tyrosine	Xenobiotics	0.55	0.01	0.02
eicosapentaenoate (EPA; 20:5n3)	Lipid	0.10	-0.01	-0.03
pyroglutamine*	Amino Acid	0.18	0.01	0.03
vanillic acid glycine	Xenobiotics	0.08	-0.01	-0.04
glycoursodeoxycholate	Lipid	0.11	-0.01	0.00
sphinganine	Lipid	0.37	0.01	-0.08
trans-urocanate	Amino Acid	0.14	0.01	0.02
S-allylcysteine	Xenobiotics	0.10	0.01	0.02
trigonelline (N'-methylnicotinate)	Cofactors and Vitamins	0.54	-0.01	0.03
phosphocholine	Lipid	0.22	-0.01	0.00
1-methylnicotinamide	Cofactors and Vitamins	0.21	0.01	0.04
palmitate (16:0)	Lipid	0.12	-0.01	0.07
2-hydroxybutyrate/2-hydroxyisobutyrate	Amino Acid	0.31	0.01	-0.01
azelate (C9-DC)	Lipid	0.25	0.01	0.02
dodecadienoate (12:2)*	Lipid	0.19	0.01	-0.03
lanthionine	Amino Acid	0.22	0.01	0.01
lactose	Carbohydrate	0.28	-0.01	-0.03
nicotinamide	Cofactors and Vitamins	0.07	-0.01	0.02
pantothenate (Vitamin B5)	Cofactors and Vitamins	0.58	-0.01	-0.11
laurate (12:0)	Lipid	0.13	-0.01	-0.02
ectoine	Xenobiotics	0.06	-0.01	0.01
dodecenedioate (C12:1-DC)*	Lipid	0.21	-0.01	0.01
methionine	Amino Acid	0.11	-0.01	0.03
3-(3-hydroxyphenyl)propionate	Xenobiotics	0.25	0.00	0.02
(N(1) + N(8))-acetylspermidine	Amino Acid	0.39	0.00	0.02
2-hydroxydecanoate	Lipid	0.20	0.00	-0.06
pregnenolone sulfate	Lipid	0.50	0.00	0.06
prolylhydroxyproline	Amino Acid	0.33	0.00	0.12
tetradecadienoate (14:2)*	Lipid	0.12	0.00	0.04
4-acetamidobutanoate	Amino Acid	0.31	0.00	0.03
N1-Methyl-2-pyridone-5-carboxamide	Cofactors and Vitamins	0.28	0.00	-0.01
cysteine-glutathione disulfide	Amino Acid	0.10	0.00	0.08
salicylate	Xenobiotics	0.00	0.00	-0.01
1-linoleoyl-GPE (18:2)*	Lipid	0.11	0.00	0.02
AMP	Nucleotide	0.28	0.00	0.12
3-hydroxystachydrine*	Xenobiotics	0.27	-0.21	0.18
dimethylglycine	Amino Acid	-0.60	-0.11	-0.06

*: putative annotation.

Table S3. List of metabolites identified with associated biochemical classes.

Super Pathway	Sub Pathway	Biochemical
Amino Acid	Alanine and Aspartate Metabolism	asparagine
		hydroxyasparagine
		N,N-dimethylalanine
	Creatine Metabolism	creatine
		creatinine
	Glutamate Metabolism	alpha-ketoglutaramate*
		glutamate
		glutamine
		pyroglutamine*
	Glutathione Metabolism	2-hydroxybutyrate/2-hydroxyisobutyrate
		cysteine-glutathione disulfide
	Glycine. Serine and Threonine Metabolism	betaine
		dimethylglycine
		glycine
		N-acetylserine
		threonine
	Guanidino and Acetamido Metabolism	4-guanidinobutanoate
	Histidine Metabolism	1-methylhistidine
		3-methylhistidine
		histidine
		N-acetyl-1-methylhistidine*
		N-acetylcarnosine
		trans-urocanate
	Leucine. Isoleucine and Valine Metabolism	1-carboxyethylvaline
		2-ketocaprylate
		3-methyl-2-oxobutyrate
		3-methylcrotonylglycine
		3-methylglutaconate
		3-methylglutarylcarnitine (2)
		alpha-hydroxyisocaproate
		beta-hydroxyisovaleroylcarnitine
		isovalerylcarnitine (C5)
		isovalerylglycine
		leucine
		N-acetylleucine
		valine
	Lysine Metabolism	2-aminoadipate
		5-hydroxylysine
		6-oxopiperidine-2-carboxylate
		hydroxy-N6,N6,N6-trimethyllysine*
		lysine
		N,N,N-trimethyl-5-aminovalerate
		N2-acetyl,N6-methyllysine
		N6,N6,N6-trimethyllysine
		N6,N6-dimethyllysine

	N6-acetyllysine
	N6-methyllysine
	pipecolate
Methionine. Cysteine. SAM and Taurine Metabolism	2-hydroxy-4-(methylthio)butanoic acid
	alpha-ketobutyrate
	cysteine
	lanthionine
	methionine
	methionine sulfone
	methionine sulfoxide
	N-acetylmethionine
	N-formylmethionine
	S-methylmethionine
	taurine
Phenylalanine Metabolism	1-carboxyethylphenylalanine
	4-hydroxyphenylacetate
	N-acetylphenylalanine
	phenylalanine
	phenyllactate (PLA)
	phenylpyruvate
Polyamine Metabolism	(N(1) + N(8))-acetylspermidine
	4-acetamidobutanoate
	5-methylthioadenosine (MTA)
	spermidine
	spermine
Tryptophan Metabolism	3-hydroxykynurenine
	6-bromotryptophan
	8-methoxykynurenate
	indoleacetate
	indolelactate
	indolepropionate
	kynurenine
	N-acetyltryptophan
	N-formylanthranilic acid
	tryptophan
	tryptophan betaine
Tyrosine Metabolism	3-(4-hydroxyphenyl)lactate (HPLA)
	3-methoxytyrosine
	tyrosine
	vanillactate
Urea cycle; Arginine and Proline Metabolism	2-oxoarginine*
	3-amino-2-piperidone
	arginine
	citrulline
	dimethylarginine (ADMA + SDMA)
	homoarginine
	homocitrulline

		hydroxyproline
		N,N,N-trimethyl-alanylproline betaine (TMAP)
		N-acetylcitrulline
		ornithine
		proline
		prolylhydroxyproline
Carbohydrate	Advanced Glycation End-product	N6-carboxymethyllysine
	Aminosugar Metabolism	glucuronate
		N-acetylglucosamine/N-acetylgalactosamine
	Disaccharides and Oligosaccharides	lactose
	Glycolysis. Gluconeogenesis. and Pyruvate Metabolism	3-phosphoglycerate
	Pentose Metabolism	arabitol/xylitol
Cofactors and Vitamins	Hemoglobin and Porphyrin Metabolism	biliverdin
	Nicotinate and Nicotinamide Metabolism	1-methylnicotinamide
		N1-Methyl-2-pyridone-5-carboxamide
		N1-Methyl-4-pyridone-3-carboxamide
		nicotinamide
		trigonelline (N'-methylnicotinate)
	Pantothenate and CoA Metabolism	pantothenate (Vitamin B5)
	Vitamin B6 Metabolism	pyridoxate
Energy	TCA Cycle	aconitate [cis or trans]
		alpha-ketoglutarate
		citraconate/glutaconate
		citrate
		succinylcarnitine (C4-DC)
Lipid	Carnitine Metabolism	carnitine
	Corticosteroids	cortisol
	Corticosteroids	cortisone
	Eicosanoid	12-HETE
	Endocannabinoid	N-oleoylserine
		N-palmitoylserine
		palmitoyl ethanolamide
	Fatty Acid Metabolism (Acyl Carnitine. Dicarboxylate)	octadecanedioylcarnitine (C18-DC)*
		octadecenedioylcarnitine (C18:1-DC)*
		suberoylcarnitine (C8-DC)
	Fatty Acid Metabolism (Acyl Carnitine. Hydroxy)	(R)-3-hydroxybutyrylcarnitine
		3-hydroxyhexanoylcarnitine (1)
	Fatty Acid Metabolism (Acyl Carnitine. Long Chain Saturated)	palmitoylcarnitine (C16)
	Fatty Acid Metabolism (Acyl Carnitine. Medium Chain)	hexanoylcarnitine (C6)
	Fatty Acid Metabolism (Acyl Carnitine. Monounsaturated)	cis-4-decenoylcarnitine (C10:1)
		myristoleoylcarnitine (C14:1)*
		oleoylcarnitine (C18)
		palmitoleoylcarnitine (C16:1)*

	undecenoylcarnitine (C11:1)
Fatty Acid Metabolism (Acyl Carnitine. Polyunsaturated)	arachidonoylcarnitine (C20:4)
	linoleoylcarnitine (C18:2)*
Fatty Acid Metabolism (Acyl Carnitine. Short Chain)	acetylcarnitine (C2)
Fatty Acid Metabolism (Acyl Glutamine)	hexanoylglutamine
Fatty Acid Metabolism (Acyl Glycine)	3-hydroxybutyroylglycine
	N-palmitoylglycine
	picolinoylglycine
Fatty Acid Metabolism (also BCAA Metabolism)	butyrylcarnitine (C4)
	propionylcarnitine (C3)
	propionylglycine (C3)
Fatty Acid. Amide	palmitamide (16:0)
Fatty Acid. Amino	2-aminoheptanoate
	2-aminooctanoate
	N-acetyl-2-aminooctanoate*
Fatty Acid. Branched	(14 or 15)-methylpalmitate (a17:0 or i17:0)
Fatty Acid. Dicarboxylate	3-carboxy-4-methyl-5-propyl-2-furanpropanoate (CMPF)
	4-hydroxy-2-oxoglutaric acid
	azelate (C9-DC)
	dodecadienoate (12:2)*
	dodecanedioate (C12)
	dodecenedioate (C12:1-DC)*
	glutarate (C5-DC)
	heptenedioate (C7:1-DC)*
	tridecenedioate (C13:1-DC)*
Fatty Acid. Dihydroxy	2R,3R-dihydroxybutyrate
Fatty Acid. Monohydroxy	13-HODE + 9-HODE
	16-hydroxypalmitate
	2-hydroxydecanoate
	2-hydroxylaurate
	2-hydroxypalmitate
	3-hydroxydecanoate
	3-hydroxyhexanoate
Glycerolipid Metabolism	glycerol 3-phosphate
Long Chain Polyunsaturated Fatty Acid (n3 and n6)	arachidonate (20:4n6)
	docosahexaenoate (DHA; 22:6n3)
	eicosapentaenoate (EPA; 20:5n3)
	linoleate (18:2n6)
	linolenate (18:3n3 or 3n6)
	nisinate (24:6n3)
	tetradecadienoate (14:2)*
Long Chain Saturated Fatty Acid	palmitate (16:0)
Lysophospholipid	1-linoleoyl-GPE (18:2)*
Medium Chain Fatty Acid	(2 or 3)-decenoate (10:1n7 or n8)
	caprate (10:0)

		caproate (6:0)
		laurate (12:0)
	Monoacylglycerol	1-myristoylglycerol (14:0)
	Phospholipid Metabolism	choline
		glycerophosphoethanolamine
		phosphocholine
		phosphoethanolamine (PE)
	Pregnenolone Steroids	21-hydroxypregnenolone disulfate
		pregnenolone sulfate
	Secondary Bile Acid Metabolism	deoxycholic acid (12 or 24)-sulfate*
		glycoursodeoxycholate
	Sphingolipid Synthesis	sphinganine
	Sphingosines	sphingosine
	Sterol	3beta-hydroxy-5-cholestenoate
		7-HOCA
Nucleotide	Purine Metabolism. (Hypo)Xanthine/Inosine containing	allantoin
		inosine 5'-monophosphate (IMP)
	Purine Metabolism. Adenine containing	1-methyladenosine
		AMP
	Purine Metabolism. Guanine containing	7-methylguanine
		guanosine
		N2,N2-dimethylguanosine
	Pyrimidine Metabolism. Cytidine containing	3-methylcytidine
		cytosine
	Pyrimidine Metabolism. Orotate containing	dihydroorotate
	Pyrimidine Metabolism. Thymine containing	5,6-dihydrothymine
	Pyrimidine Metabolism. Uracil containing	5,6-dihydrouridine
		uracil
Peptide	Dipeptide	phenylalanylhydroxyproline*
		prolylglycine
		valylleucine
	Gamma-glutamyl Amino Acid	gamma-glutamyl-2-aminobutyrate
		gamma-glutamylalanine
		gamma-glutamylglutamate
		gamma-glutamylglutamine
		gamma-glutamylglycine
		gamma-glutamylhistidine
		gamma-glutamylphenylalanine
		gamma-glutamylthreonine
		gamma-glutamyltyrosine
		gamma-glutamylvaline
Xenobiotics	Benzoate Metabolism	3-(3-hydroxyphenyl)propionate
		3-hydroxyhippurate
		3-methoxycatechol sulfate (2)
		3-phenylpropionate (hydrocinnamate)
		4-hydroxyhippurate
	Chemical	1,2,3-benzenetriol sulfate (2)

		6-hydroxyindole sulfate
		ectoine
		iminodiacetate (IDA)
		O-sulfo-L-tyrosine
		thioproline
	Drug - Analgesics. Anesthetics	2-hydroxyacetaminophen sulfate*
		2-hydroxyibuprofen
		3-(N-acetyl-L-cystein-S-yl) acetaminophen
		lidocaine
	Drug - Antibiotic	metronidazole
	Drug - Cardiovascular	metoprolol acid metabolite*
	Drug - Psychoactive	citalopram propionate*
	Drug - Topical Agents	salicylate
	Food Component/Plant	2-piperidinone
		3.4-methyleneheptanoate
		3-formylindole
		3-hydroxystachydrine*
		3-indoleglyoxylic acid
		cinnamoylglycine
		dihydroferulate
		ergothioneine
		erythritol
		gluconate
		homostachydrine*
		indolin-2-one
		methyl glucopyranoside (alpha + beta)
		methyl indole-3-acetate
		N-(2-furoyl)glycine
		N-acetyltheanine
		quinate
		S-allylcysteine
		stachydrine
		tartarate
		theanine
		thymol sulfate
		vanillic acid glycine
	Tobacco Metabolite	cotinine
		hydroxycotinine
	Xanthine Metabolism	1,3,7-trimethylurate
		caffeine
		paraxanthine
		theobromine
Partially Characterized Molecules		glutamine conjugate of C6H10O2 (1)*
		glutamine conjugate of C6H10O2 (2)*
		glycine conjugate of C10H14O2 (1)*

*: putative annotation.

Table S4. PLS-R model of metabolites reported. Pathway indicates which metabolites from the mothers were used in the model. w24=plasma from mothers at 24 weeks of pregnancy (N=664). w1=plasma form mothers 1 week postpartum (N=661). LV=number of latent variables included in the model.

Biochemical	Pathway	Mothers	LV	R^2 Cal	R^2 CV	RMSE Cal	RMSE CV
3-carboxy-4-methyl-5-propyl-2-furanpropanoate	all	w24	2	0.31	0.00	$5.22X10^{-4}$	$7.75X10^{-3}$
	Lipids	w24	2	0.23	0.10	$5.50X10^{-4}$	$6.00X10^{-4}$
	Fatty Acid. Dicarboxylate	w24	2	0.25	0.20	$5.40X10^{-4}$	$5.60X10^{-4}$
	all	w1	2	0.60	0.49	$3.90X10^{-4}$	$4.40X10^{-4}$
	Lipids	w1	2	0.59	0.53	$3.90X10^{-4}$	$4.20X10^{-4}$
	Fatty Acid. Dicarboxylate	w1	4	0.76	0.74	$3.00X10^{-4}$	$3.20X10^{-4}$
tryptophan betaine	all	w24	4	0.62	0.18	$4.60X10^{-4}$	$7.12X10^{-4}$
	Amino Acids	w24	4	0.56	0.35	$4.92X10^{-4}$	$6.06X10^{-4}$
	Tryptophan Metabolism	w24	4	0.47	0.43	$5.42X10^{-4}$	$5.63X10^{-4}$
	all	w1	2	0.41	0.12	$6.18X10^{-4}$	$7.82X10^{-4}$
	Amino Acids	w1	2	0.60	0.44	$5.08X10^{-4}$	$6.05X10^{-4}$
	Tryptophan Metabolism	w1	2	0.70	0.68	$4.05X10^{-4}$	$4.18X10^{-4}$
ergothioneine	all	w24	11	0.89	0.20	$4.60X10^{-5}$	$1.37X10^{-4}$
	Xenobiotics	w24	3	0.51	0.31	$9.60X10^{-5}$	$1.16X10^{-4}$
	Food component/ plant	w24	3	0.53	0.45	$9.40X10^{-5}$	$1.02X10^{-4}$
	all	w1	2	0.37	0.12	$1.09X10^{-4}$	$1.36X10^{-4}$
	Xenobiotics	w1	3	0.49	0.20	$9.78X10^{-5}$	$1.40X10^{-4}$
	Food component/ plant	w1	3	0.51	0.43	$9.60X10^{-5}$	$1.04X10^{-4}$
N6-methyllysine	all	w24	3	0.59	0.09	$1.85X10^{-5}$	$3.63X10^{-5}$
	Amino Acids	w24	3	0.45	0.29	$2.14X10^{-5}$	$2.43X10^{-5}$
	Lysin Metabolism	w24	4	0.38	0.35	$2.26X10^{-5}$	$2.31X10^{-5}$
	all	w1	2	0.42	0.15	$2.22X10^{-5}$	$2.74X10^{-5}$
	Amino Acids	w1	2	0.38	0.27	$2.50X10^{-5}$	$2.49X10^{-5}$
	Lysin Metabolism	w1	2	0.37	0.35	$2.30X10^{-5}$	$2.35X10^{-5}$
N,N,N-trimethyl-5-aminovalerate	all	w24	2	0.19	0.05	$1.20X10^{-3}$	$1.30X10^{-3}$
	Amino Acids	w24	2	0.29	0.13	$1.10X10^{-3}$	$1.20X10^{-3}$
	Lysin Metabolism	w24	2	0.31	0.28	$1.10X10^{-3}$	$1.10X10^{-3}$
	all	w1	2	0.35	0.07	$1.10X10^{-3}$	$1.40X10^{-3}$
	Amino Acids	w1	3	0.43	0.20	$9.90X10^{-4}$	$1.20X10^{-3}$
	Lysin Metabolism	w1	3	0.32	0.29	$1.10X10^{-3}$	$1.20X10^{-3}$
stachydrine	all	w24	2	0.33	0.12	$4.35X10^{-3}$	$5.15X10^{-3}$

	Xenobiotics	w24	2	0.26	0.14	4.58X10^{-3}	5.00X10^{-3}
	Food component/ plant	w24	2	0.21	0.14	4.73X10^{-3}	4.98X10^{-3}
	all	w1	3	0.51	0.23	3.73X10^{-3}	4.82X10^{-3}
	Xenobiotics	w1	2	0.34	0.20	4.33X10^{-3}	4.83X10^{-3}
	Food component/ plant	w1	2	0.30	0.25	4.45X10^{-3}	4.62X10^{-3}
**homostachydrine***	all	w24	1	0.19	0.06	3.00X10^{-5}	3.00X10^{-5}
	Xenobiotics	w24	2	0.23	0.05	3.00X10^{-5}	3.70X10^{-5}
	Food component/ plant	w24	2	0.16	0.07	3.20X10^{-5}	3.40X10^{-5}
	all	w1	1	0.20	0.04	3.10X10^{-5}	3.50X10^{-5}
	Xenobiotics	w1	1	0.11	0.03	3.20X10^{-5}	3.60X10^{-5}
	Food component/ plant	w1	2	0.27	0.12	3.00X10^{-5}	3.30X10^{-5}
**homoarginine**	all	w24	1	0.18	0.03	2.26X10^{-5}	2.53X10^{-5}
	Amino Acids	w24	2	0.36	0.17	2.00X10^{-5}	2.31X10^{-5}
	Urea cycle; Arginine and Proline Metabolism	w24	3	0.29	0.25	2.11X10^{-5}	2.16X10^{-5}
	all	w1	2	0.32	0.02	2.06X10^{-5}	3.13X10^{-5}
	Amino Acids	w1	2	0.27	0.11	2.12X10^{-5}	2.37X10^{-5}
	Urea cycle; Arginine and Proline Metabolism	w1	3	0.21	0.16	2.21X10^{-5}	2.28X10^{-5}
**paraxanthine**	all	w24	2	0.38	0.00	1.50X10^{-4}	3.70X10^{-3}
	Xenobiotics	w24	2	0.31	0.00	1.60X10^{-4}	1.10X10^{-2}
	Xanthine Metabolism	w24	2	0.23	0.21	1.70X10^{-4}	1.70X10^{-4}
	all	w1	2	0.34	0.06	1.50X10^{-4}	2.00X10^{-4}
	Xenobiotics	w1	2	0.29	0.01	1.60X10^{-4}	3.70X10^{-4}
	Xanthine Metabolism	w1	2	0.21	0.16	1.70X10^{-4}	1.80X10^{-4}
**cotinine**	all	w24	2	0.38	0.00	2.36X10^{-5}	3.70X10^{-3}
	Xenobiotics	w24	2	0.45	0.01	2.21X10^{-5}	1.00X10^{-3}
	all	w1	2	0.32	0.06	2.40X10^{-5}	2.97X10^{-5}
	Xenobiotics	w1	2	0.22	0.04	2.57X10^{-5}	3.42X10^{-5}
**caffeine**	all	w24	1	0.27	0.09	8.54X10^{-1}	9.70X10^{-1}
	Xenobiotics	w24	1	0.19	0.07	9.00X10^{-1}	1
	Xanthine Metabolism	w24	1	0.21	0.20	8.80X10^{-1}	8.90X10^{-1}
	all	w1	1	0.24	0.07	8.70X10^{-1}	9.80X10^{-1}
	Xenobiotics	w1	1	0.15	0.07	9.10X10^{-1}	9.70X10^{-1}
	Xanthine Metabolism	w1	1	0.01	0.00	9.90X10^{-1}	1

*: putative annotation.

Table S5. Bootstrapped replication results for top 11 metabolites exhibiting vertical transfer. R_{m1-c} refers to the observed Pearson correlations, with bootstrapped 95% confidence bounds. # Discovery shows the discovery rate at the level of $R_{m1-c} > 0.3$, # replication shows the rate of the out of bag replication results at level $R_{m1-c} > 0.3$ as well as plain significance $p < 0.05$. From a total of 1000 bootstraps.

BIOCHEMICAL	R_{m1-c}	# Discovery	# replication (r>0.3)	# replication (p < 0.05)
CMPF	0.87 (0.84-0.89)	1000 (100%)	1000 (100%)	1000 (100%)
tryptophan betaine	0.82 (0.71-0.86)	1000 (100%)	1000 (100%)	1000 (100%)
ergothioneine	0.68 (0.61-0.74)	1000 (100%)	1000 (100%)	1000 (100%)
N6-methyllysine	0.6 (0.54-0.65)	1000 (100%)	1000 (100%)	1000 (100%)
N,N,N-trimethyl-5-aminovalerate	0.54 (0.48-0.6)	1000 (100%)	1000 (100%)	1000 (100%)
stachydrine	0.5 (0.43-0.56)	1000 (100%)	1000 (100%)	1000 (100%)
homostachydrine*	0.43 (0.31-0.54)	988 (99%)	941 (95%)	987 (100%)
homoarginine	0.42 (0.34-0.49)	997 (100%)	985 (99%)	997 (100%)
paraxanthine	0.4 (0.32-0.47)	995 (100%)	974 (98%)	995 (100%)
cotinine	0.36 (0.26-0.66)	943 (94%)	771 (82%)	932 (99%)
caffeine	0.35 (0.26-0.44)	867 (87%)	677 (78%)	867 (100%)

*: putative annotation.

Figure S1. Robustness analysis for the transfer results given the fish oil intervention.

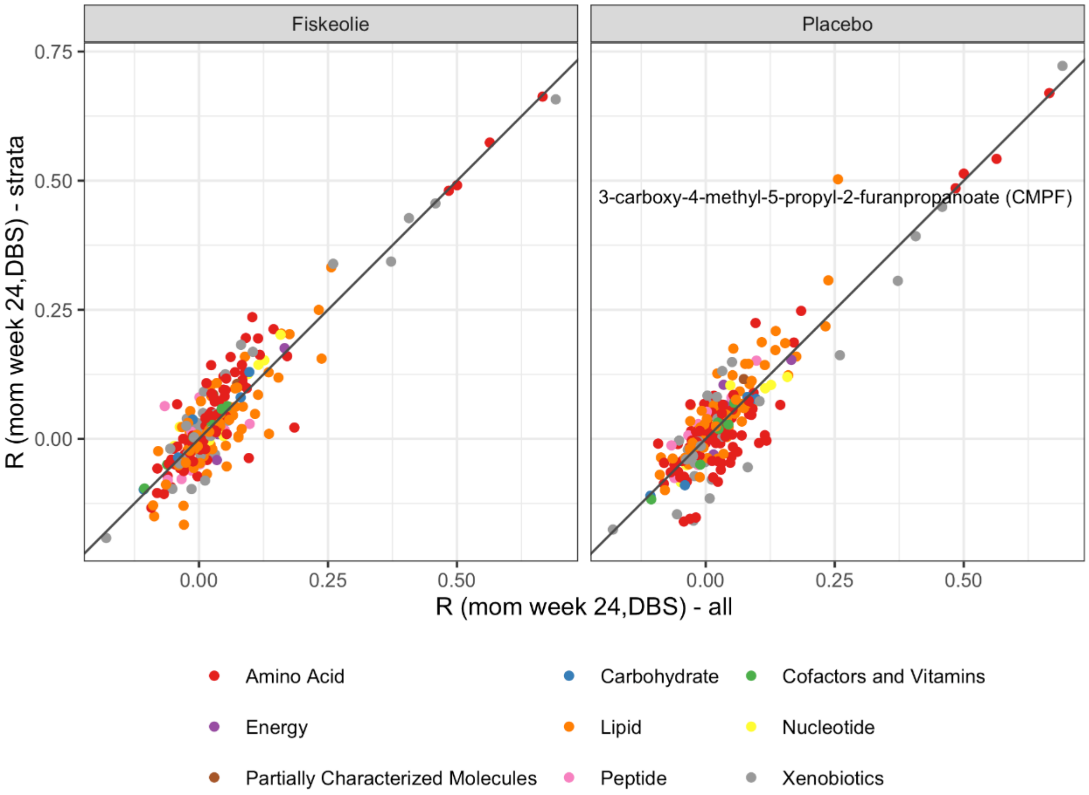

Figure S2. Child metabolite level from DBS as function of maternal week 24 mid pregnancy (red) and one week postpartum (blue) blood levels of the 11 metabolites with strong transfer merits ($R_{m1\text{-}c} > 0.3$). The correlations denoted R and rho refers to Pearson and Spearman correlation coefficients respectively.

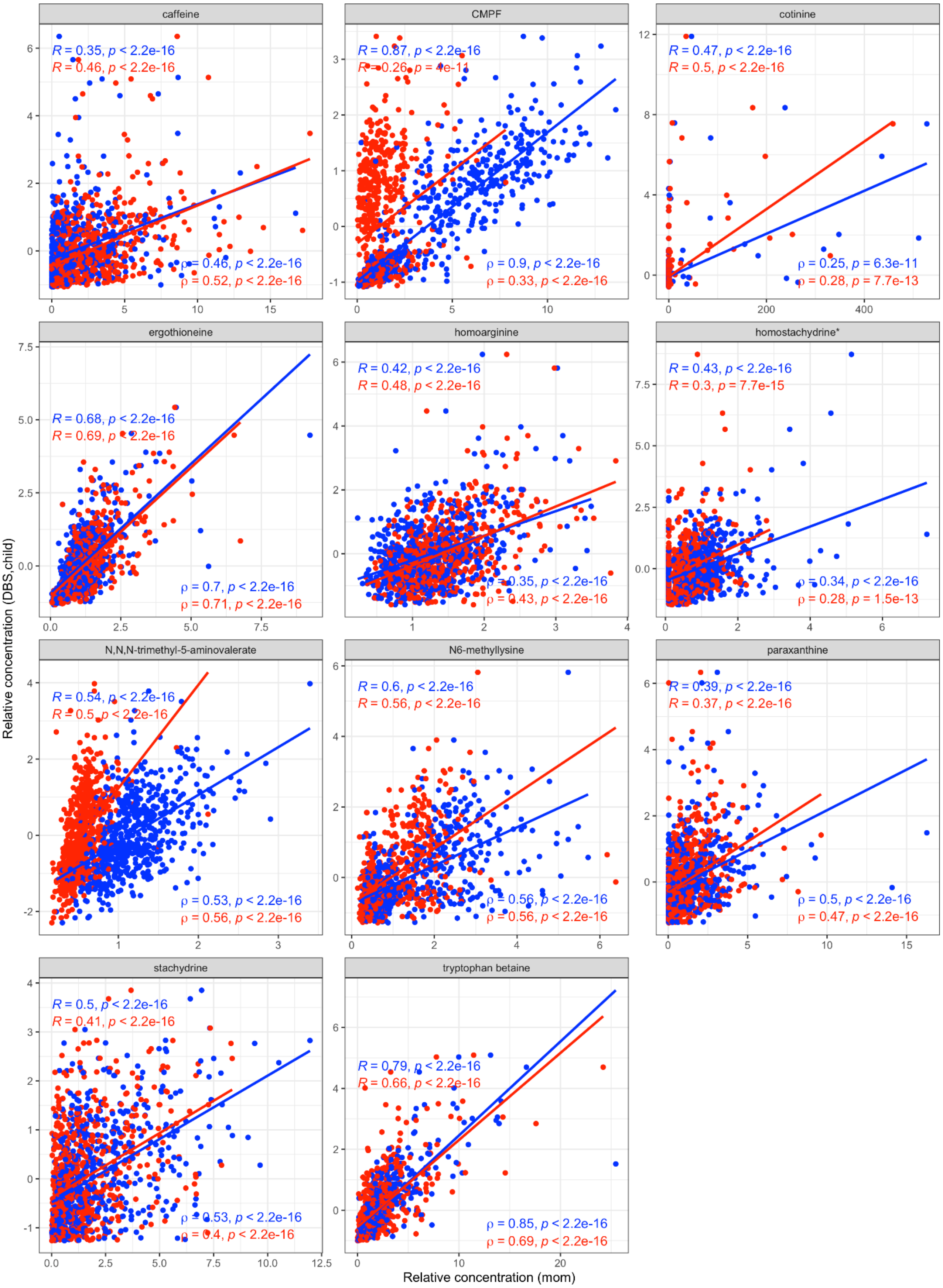

Figure S3. Maternal levels of one week postpartum samples versus week 24 mid pregnancy samples of the 11 metabolites with strong transfer merits ($R_{m1\text{-}c} > 0.3$). The correlations denoted R and rho refers to Pearson and Spearman correlation coefficients respectively.

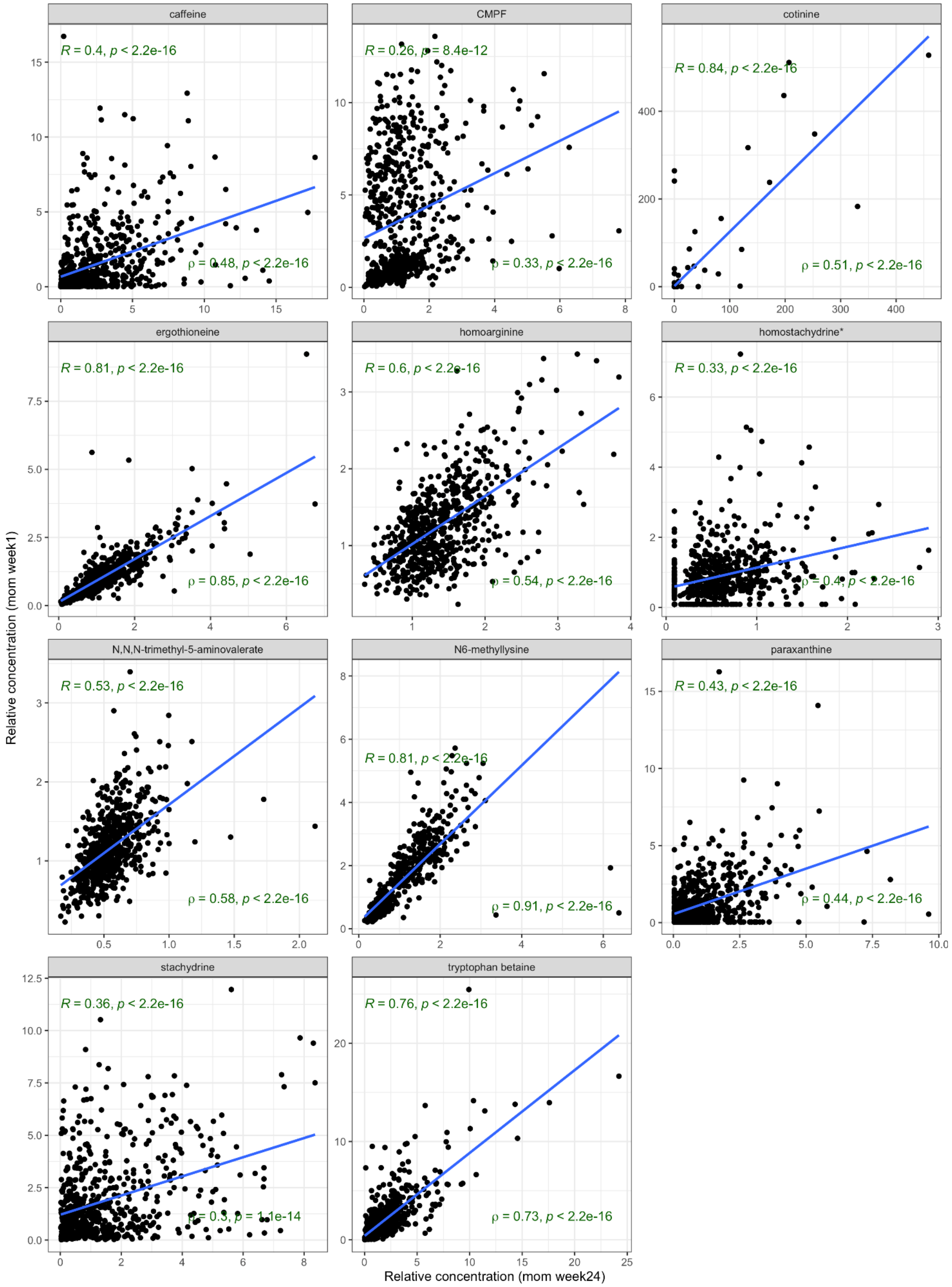

Figure S4. PCA loading plot of plasma from mothers at 24 weeks of pregnancy. Loadings were color-coded by correlation $R_{m1\text{-}c}$.

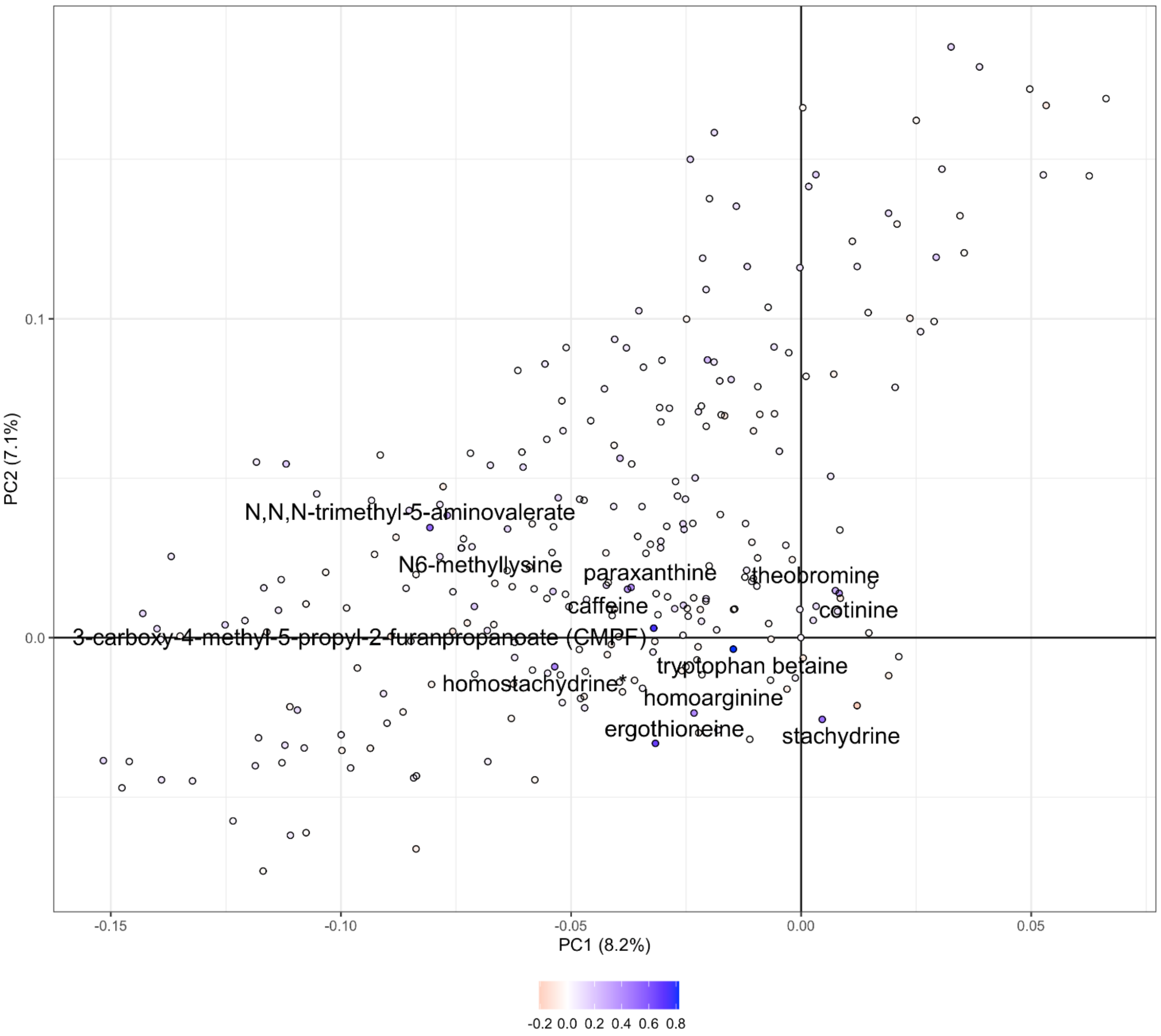

Figure S5. PCA loading plot of plasma from mothers 1 week postpartum. Loadings were color-coded by correlation $R_{m1\text{-}c}$.

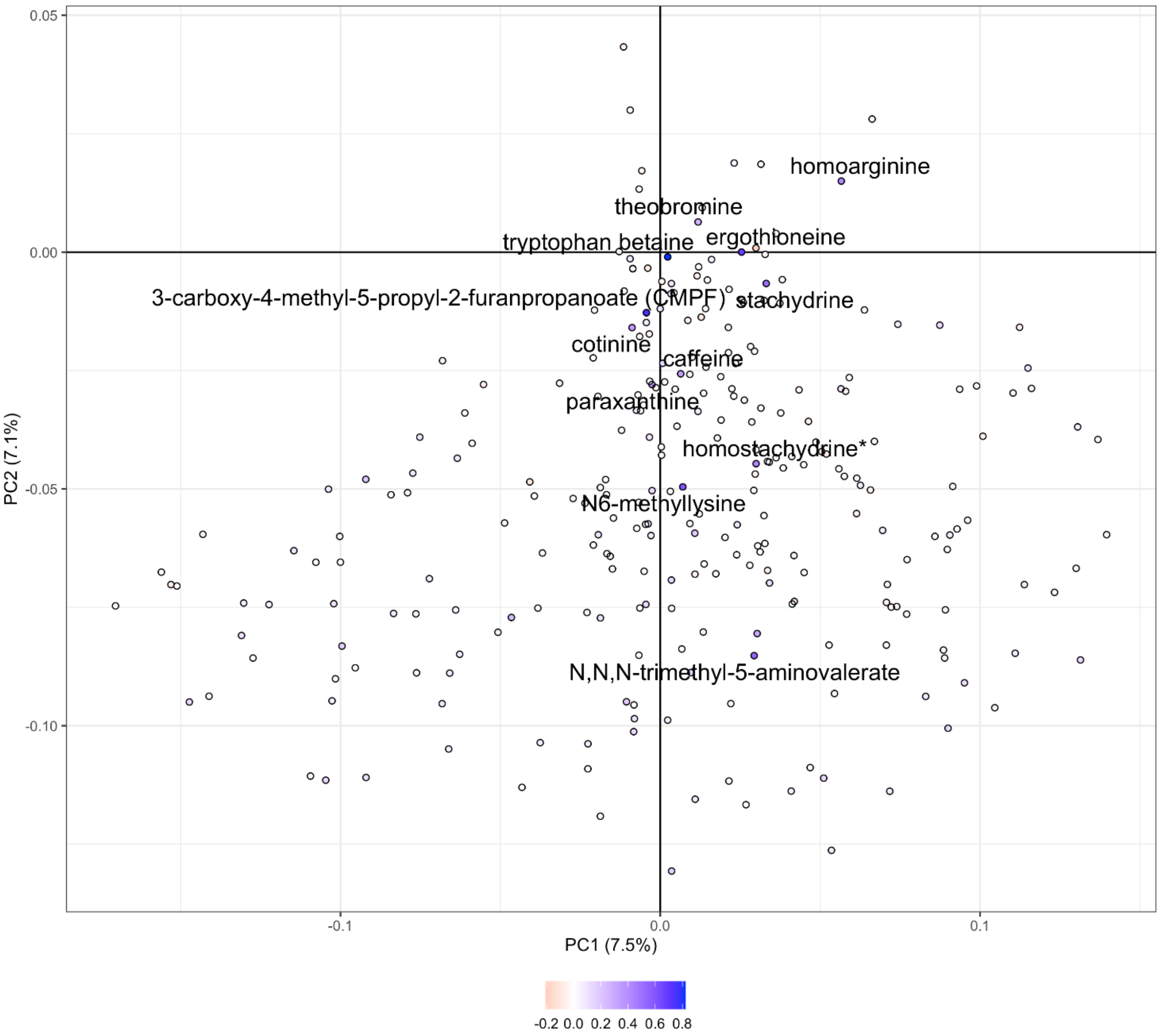

Figure S6. PCA loading plot of DBS samples from newborns. Loadings were color-coded by correlation R_{m1-c}.

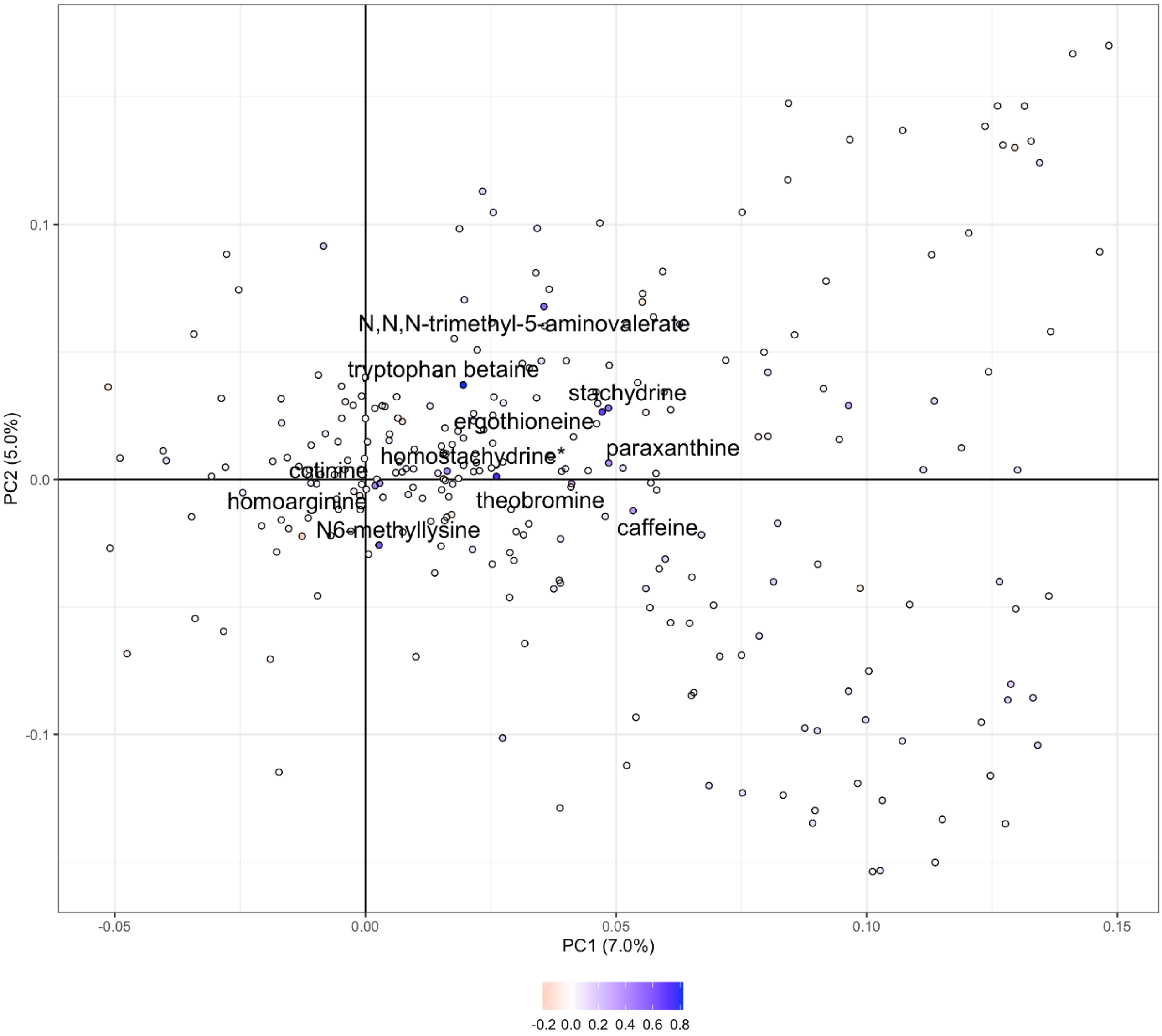

In addition to the PCA analysis the correlation between the metabolites with transfer statistics $R_{m1\text{-}c} > 0.3$ (denoted within in Figure S7) is compared with the correlations between metabolites with $R_{m1\text{-}c} > 0.3$ and metabolites with $R_{m1\text{-}c} \leq 0.3$ (denoted between in Figure S7). This analysis is in agreement with the interpretation of the loadings plot, namely that there is not a particular pattern characterizing the metabolites exhibiting strong transfer statistics.

Figure S7. Metabolite to metabolite correlation computed within each time point and partitioned into within transferred metabolites ($R_{m1\text{-}c} > 0.3$) and between none-to weak transferred ($R_{m1\text{-}c} \leq 0.3$) and transferred ($R_{m1\text{-}c} > 0.3$) metabolites.

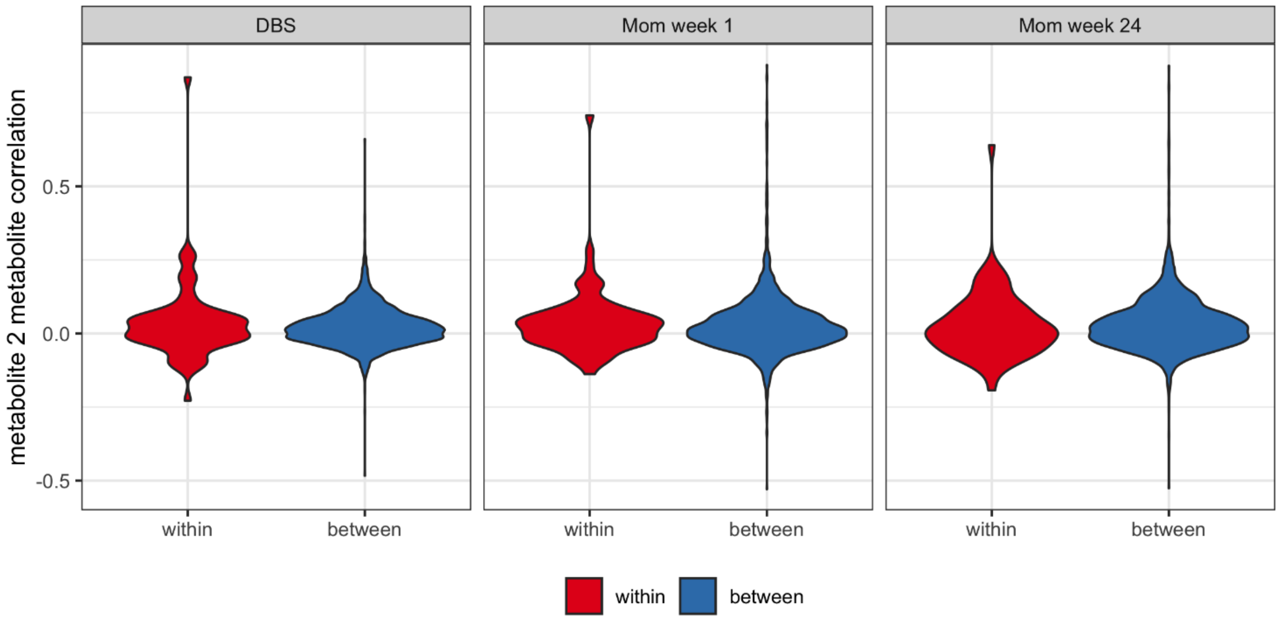

Table S6. Descriptive (median and IQR) for metabolite to metabolite correlations within each time point, and partitioned into within transferred metabolites ($R_{m1\text{-}c} > 0.3$) and between none-to weak transferred ($R_{m1\text{-}c} \leq 0.3$) and transferred ($R_{m1\text{-}c} > 0.3$) metabolites.

time	between	within
DBS	0.02 (-0.02;0.06)	0.02 (-0.03;0.06)
Mom week 1	0.01 (-0.03;0.06)	0.02 (-0.03;0.06)
Mom week 24	0.02 (-0.03;0.07)	0.01 (-0.05;0.08)
